# Two Cases of Lithontripsy, by the Late Geo. McClellan, M. D.

**Published:** 1849-09

**Authors:** J. H. B. McClellan


### MEDICAL EXAMINER,

AND

### RECORD OF MEDICAL SCIENCE.

NEW SERIES.—NO. LVII.—SEPTEMBER, 1849.

### ORIGINAL COMMUNICATIONS.

*Two cases of Lithontripsy, by the late* Geo. McClellan, M. D. Reported by his son, J. H. B. McClellan, M. D., from notes taken at the time by E. R. Mayer, M. D.

Mr. Miller, æt. 22, of Kingston, Canada, carpenter by trade, came to Dr. McClellan, May 10th, 1843, suffering from a very large calculus in the bladder. He attributed its formation to his having, a few years ago, mischievously put a kidney bean into the urethra. After its insertion he forgot the circumstance, and thinks it must have worked its way into the bladder.

Upon sounding him, the stone appeared to be hard and very large. He has never passed any particles. Mr. M. is a very nervous and timid man, and has taken a great deal of opium to relieve the pain. He has a great horror of cutting, and wishes to be relieved, if possible, by the crushing operation. Under these circumstances, Dr. McC. consented to use the lithontriptic instruments, which, he remarked, are more painful than cutting instruments, though less dangerous, and are more applicable to old age.

Some difficulty was apprehended, from Mr. Miller's extreme irritability—he cannot urinate in the erect position, without, at the same time, discharging his fæces, a common occurrence with those having calculi, owing to the tenesmus from the irritability of the bladder.

*May* 11*th*. Introduced a small Jacobson instrument, but found that it did not catch the stone readily. Finding the urethra sufficiently large, he introduced a larger one of Heurteloup's, with which he immediately caught the stone. (No positive rule can be laid down in regard to the choice of these instruments; sometimes one being applicable, sometimes the other.) By five or six efforts he succeeded in reducing a portion of the stone (which must have been three inches in diameter) into a number of fragments. It was hard, and apparently shelled off in strata. The patient bore the operation very well, suffering only from the irritability of the bladder, which occasionally contracted and ejected the urine.

Notwithstanding the utmost care, the Dr. could not disengage a few fragments from between the blades, and although the instrument could be readily introduced, he had to use some gentle force in withdrawing it, a common and sometimes unavoidable occurrence in this operation. After the operation, an opiate was administered, and he was directed to drink freely of an infusion of Erigeron Philadelphicum.

*May* 12*th*. Repeated the operation with a small Heurteloup's instrument, and crushed several large fragments. The patient rather more irritable, from pain in the bladder and urethra. Passed a few fragments, and amongst them a piece a third of an inch in diameter in one direction.

It will require a number of operations to crush the stone entirely, and even when crushed, it will probably require a long time to effect a cure, the patient not being able at present to discharge his water in an upright position.

*May* 15*th*. Repeated the operation, and crushed a large fragment.

*May* 17*th and* 19*th*. Notwithstanding all difficulties, crushed several large fragments. Quantities of small grit, and some large pieces have come away, giving him, as yet, but little pain.

Nitro-muriatic acid being administered, in a few days greatly cleared and made neutral his urine, which before abounded in sediment, and was strongly ammoniacal. He is still taking the erigeron, with a little morphia. The calculus is white, stratified, and pretty hard; and I have ascertained it to be the triple phosphate of magnesia, ammonia and lime.

*May 20th.* Repeated the crushing. His urethra and bladder were less irritable than before; very little pain was produced, and no blood discharged as in previous instances. On this occasion, the Dr. seized the stone by its long diameter, and apparently broke it into several fragments, while in the previous attempts he had merely chipped off small pieces; the stone being so large as to prevent the whole of it being engaged in the instrument at once. The patient is less irritable, passes his urine more readily in a position inclining to the erect, and without discharging his fæces; while the expulsion of the fragments through his urethra is attended with much less pain, and proceeds quite rapidly.

*May 21st.* Operation repeated.

*May 22d.* Used Jacobson's small instrument in order to comminute some of the smaller fragments. Each time the instrument is removed, it is clogged up with gritty particles, and is with difficulty drawn from the meatus. In this way alone, quite a quantity of the stone has been withdrawn from the bladder. The patient has become accustomed to the use of the instrument, his urethra and bladder are less irritable, and he suffers but little pain during the operation, and generally, except during the expulsion of the fragments.

*June 1st.* By repeated operations the calculus is at length reduced to a comparatively small size. During the past week he has voided with his urine over ℥ij. of fragments, most of them larger than a pea, and some still larger. Owing to imprudence in diet, and other causes, he has been seized during this time with bilious vomiting, hiccough, and other unpleasant symptoms, which were checked, however, by the exhibition of hydrar. chloridi mit. gr. xxv., and the general irritability of his system thus dissipated. The Dr. succeeded this morning, with a middle sized Heurteloup's instrument, in grasping the stone and breaking off some large pieces. Deeming, from the increased resistance he experienced, that he had seized the nucleus of the stone, the Dr. continued turning the screw, when suddenly a loud crack was heard, caused by the breaking of the inner blade of the instrument.

The accident excited no alarm, however, as it will probably be passed by the urethra. On three occasions this has happened to Dr. McC., with this result.

*June 3d.* Repeated the operation, and crushed a large quantity, without, however, coming in contact with the broken blade. A piece of stone lodged high up in the meatus, which, however, did not interfere with his urination.

*June 5th.* In discharging his fæces, a piece of the calculus came out and lodged in the urethra, about an inch behind the corona glandis. The piece was large, angular, and defied all attempts to dislodge it with the polypus forceps; nor could it have been extricated without lacerating the urethra to a great degree. The Dr. therefore cut down upon it through the corpus spongiosum, and readily removed it.

*June 19th.* Since the last date the Dr. has operated two or three times, and there remain but few pieces of any size. A large quantity of detritus can be felt by the catheter in the bas-fond of the bladder.

Every day he passes small fragments, and a good deal of this detritus. The larger pieces are extruded without inconvenience through the fistulous opening in the lower part of the penis—which has been kept open, there being no danger of infiltration. His general health is the same, while he appears less irritable, and expresses his consciousness of relief, and of a diminution of the heavy, oppressive feeling produced by the calculus. The blade of the lithotrite still remains.

He still continues the use of erigeron tea, nitro-muriatic acid, opium, &c.

*June 28th.* Repeated the operation, and crushed the remaining pieces of any size. The patient was directed to sit up, and discharge his urine in the erect position. (He had been frequently urged to do this before, but always positively refused, asserting the impossibility of the attempt, and evincing considerable nervousness and irritability.) This time, however, he assented, and after many attempts to discharge his urine, which were ineffectual on account of his fear and conviction of the impossibility of the attempt, he succeeded at last in producing a full stream, containing about a handful of small fragments; and followed by the broken blade, which was extruded without much effort or pain. It was much rusted, but showed no appearance of concretions upon it. Upon sounding him, it was ascertained that but little of the stone remained, and that, probably, in a state of extreme division.

The patient is much relieved, and expresses strong hopes of recovery.

*July 19th.* The patient during the past weeks has been passing very fine detritus. Expresses himself entirely free from pain, and believes there is no foreign substance in his bladder. He urinates readily; either standing or sitting; is very much emaciated, however, and reduced almost to a skeleton. A week or two ago he was attacked with diarrhœa, pain in the breast, and profuse expectoration, so much so that phthisis was feared as a very natural consequence of his present condition. He is, however, gradually recovering.

*August 25th.* The Dr., since the last date, has crushed two or three small fragments, and the patient is now in good condition. He enjoys good appetite, and has not suffered from the least symptoms of stone for the last few weeks. He urinates in the erect position, and his urine is healthy and limpid. He considers himself perfectly cured, and has sought and obtained work as a carpenter.

The fragments taken from him nearly fill a f.℥vj. vial.

Case 2. Mr. P., of New Jersey, upwards of sixty, April 27, 1843. In the first operation Jacobson's instrument was used, and subsequently Heurteloup's; the latter being generally preferred as being more easy to find the stone with.

At the first operation, the instrument was introduced without difficulty, the stone found and crushed. It proved to be a mulberry calculus, (oxalate of lime,) and did not fall to pieces as the other varieties commonly do.

*May 10th.* Between this and the last date, Dr. McC. has crushed five or six large fragments, the detritus of which has been passed. To-day crushed several fragments. He leaves for home to-day.

*May 16th.* Mr. P. returned from Jersey, to be examined finally. One or two small fragments were crushed into powder, and discharged during the day. He declares himself perfectly free from all unpleasant sensations—in a word, perfectly cured.

Stone in the bladder may be distinguished from inflamed or irritable bladder, (which sometimes resembles it,) by the patient in the former affection feeling most easy when the bladder is full, in the latter, when it is empty.

